# Barriers and facilitators to depression care among Latino men in a primary care setting: a qualitative study

**DOI:** 10.1186/s12875-024-02275-x

**Published:** 2024-01-20

**Authors:** Nathan Swetlitz, Ladson Hinton, Morgan Rivera, Mishen Liu, Anna Claire Fernandez, Maria E Garcia

**Affiliations:** 1 UC Berkeley, UCSF Joint Medical Program, University of California, Berkeley, Berkeley, CA USA; 2 School of Medicine, University of California, San Francisco, San Francisco, CA USA; 3 University of California, Davis, Davis, CA USA; 4 University of California, Berkeley, Berkely, CA USA; 5 Multiethnic Health Equity Research Center, Division of General Internal Medicine, Department of Medicine, University of California, San Francisco, CA USA; 6 Department of Epidemiology and Biostatistics, University of California, San Francisco, CA USA; 7 Partnerships for Research in Implementation Science for Equity, University of California, San Francisco, San Francisco, CA USA

**Keywords:** Depression, Primary care, Masculinity, Barriers and facilitators to care, Latino men

## Abstract

**Background:**

In the United States, Latinos face a wide array of cultural and structural barriers to accessing and utilizing mental health care. Latino men specifically are at high risk of receiving inadequate mental health care, possibly due to additional obstacles they experience that are related to masculinity. Among men more generally, greater adherence to emotional control and self-reliance is associated with higher depression severity and less depression help-seeking. Men experience more stigma toward depression and help-seeking and are less likely to be diagnosed with depression than women. However, Latino men’s barriers and facilitators to depression care remain largely unexplored. The objective of this study was to examine barriers and facilitators to depression care that are related to masculinity among English- and Spanish-speaking Latino men in a primary care setting.

**Methods:**

We used convenience and purposive sampling to recruit primary care patients who self-identified as Latino men, spoke English or Spanish, and screened positive for depressive symptoms on the Patient Health Questionnaire-2 or had a history of depression. Semi-structured interviews were conducted between December 2020 and August 2021. The interview guide examined views and experiences of depression, masculinity, and barriers and facilitators to engaging in depression care. Utilizing consensual qualitative research and thematic analysis informed by modified grounded theory, barriers and facilitators to depression care were identified.

**Results:**

We interviewed thirteen participants who varied in English proficiency, education, income, and country of origin. Barriers and facilitators were placed into three domains—Self-Recognition of Depression, Seeking Help for Depression, and Depression Diagnosis and Treatment. Participants described aspects of masculinity as barriers (emotional control and pressure to provide), facilitators (honesty, courage, collaboration, practicality, and responsibility), or both (self-reliance and autonomy).

**Conclusions:**

Masculinity influences barriers and facilitators for depression care among Latino men at the levels of self-recognition, seeking help, and diagnosis and treatment. Clinicians may promote Latino men’s engagement in depression care by understanding patients’ values and framing depression care as affirming masculinity. Providing education to primary care physicians and other healthcare professionals on gender and depression and addressing structural barriers are essential to providing access to all who need depression care.

**Supplementary Information:**

The online version contains supplementary material available at 10.1186/s12875-024-02275-x.

## Introduction

In the United States, Latino individuals experience a variety of mental health disparities compared to non-Latino white (hereafter ‘white’) individuals. While Latinos suffer from psychiatric conditions at the same or greater rates as whites, they have less access to and utilization of mental health care [1–6]. Latinos face cultural barriers to engaging in mental health care including stigma and different explanatory models for psychiatric disorders [4, 7–9]. They also experience structural barriers including prohibitive costs, insufficient insurance coverage, lack of linguistically and culturally concordant care, and transportation [4, 7–12]. Latino men may experience particularly high risks for unmet mental health care. In one study, Latino men with low English proficiency had 2.5 times the likelihood of unmet mental health needs compared to Latina women [1]. Depression is the most prevalent mental illness in the United States, severely decreasing quality of life [13–15]. While depression rates have increased in recent years, levels of depression treatment have remained steady, suggesting depression is widely undertreated [13]. In the United States, Latinos are less likely to use and more likely to discontinue antidepressants than white individuals [15–17]. Compared to older white men, older Mexican American men with high English proficiency are 4 times less likely to receive depression treatment, and those with low English proficiency are ten times less likely [18]. Latino men may also experience barriers to depression care that are related to masculinity—the social and cultural roles, expectations, and ideas concerning what it means to be a man [19, 20]. We define depression care as healthcare professionals providing depression screenings, diagnoses, referrals, psychotherapy, or antidepressants to people experiencing depressive symptoms. We define depression treatment more narrowly as healthcare professionals prescribing antidepressants or providing psychotherapy to people experiencing depressive symptoms.

Increasing evidence suggests men are underdiagnosed with depression compared to women, despite dying from suicide at higher rates [21–24]. While pathways to dying by suicide are complex and multifactorial, over 90% of completed suicides are associated with mental illness, the most common of which is major depressive disorder (MDD) [25–29]. People with MDD have at least a five times greater risk of attempting suicide compared to the general population [25, 30, 31]. Although epidemiological data consistently indicate men are less likely to suffer from depression, social beliefs about masculinity may obscure recognition, diagnosis, and treatment of men’s depression [21, 22, 32–38]. Greater adherence to traditional Western masculine norms like emotional control and self-reliance has been associated with less depression help-seeking and higher depression severity among men [20–22, 36, 37, 39, 40]. Increased stigma among men regarding depression, seeking medical and psychological help, and depression treatment is correlated with lower rates of seeking help for depression [22, 39, 41–45]. Clinicians may recognize depression less often in men than women, even when presenting with the same symptoms. Researchers attribute this disparity partially to clinicians having different expectations for their patients based on gender norms [45, 46]. For example, it may be more difficult for clinicians to identify depressive symptoms in men—whom they expect to maintain emotional control—and easier for them to identify depressive symptoms in women—whom they expect to exhibit vulnerability and emotional lability [46, 47]. 

Prior studies, primarily examining white men’s experiences, [22, 36, 48–50] may not reflect the unique challenges and opportunities Latino men have in engaging with depression care. Previous research has also focused on the negative effects masculine norms may have on men’s seeking and receiving depression care. While researchers have started exploring facilitators shaped by masculinity, attention is often secondary or speculative, providing few opportunities to leverage facilitators to depression care shaped by masculinity [48, 50–53]. Furthermore few studies have examined barriers and facilitators for men receiving depression care in primary care settings, limiting the applicability of existing data to improving access to and engagement with depression care in these settings, where most individuals receive depression care [22, 50, 54–56]. 

Latino men’s specific experiences, barriers, and facilitators to depression care remain largely unexplored [39, 49, 57, 58]. Latino men’s understanding of and experiences associated with masculinity may also be substantively different than those of white men. Culturally specific formulations of Latino masculinity include caballerismo and machismo. Caballerismo is often described as the prosocial components of Latino masculinity, including courage, nurturing character, responsibility, family-centeredness and integrity [59–61]. Conversely, machismo is usually defined as chauvinistic or antisocial components of Latino masculinity, including dominance, aggression, and sexism [59–61]. Although their conceptions and realizations of masculinity vary greatly, Latino men often encounter stereotypes that equate Latino masculinity with machismo, portraying all Latino men as aggressive, threatening, sexist, and hypersexualized [61–64]. Latino men’s experiences with depression and access to depression care may be uniquely shaped both by their own relationship with Latino masculinity, and also by how clinicians perceive Latino men.

This study examines barriers and facilitators to seeking, receiving, and utilizing depression care among English- and Spanish-speaking Latino men with depressive symptoms in primary care settings, focusing on how masculinity may affect accessibility of and engagement in depression care.

## Methods

### Setting, participants, and study design

To identify barriers and facilitators derived from participants’ experiences, views, understandings, and identities, we utilized the consensual qualitative research (CQR) methodology and thematic analysis, informed by modified grounded theory [65–67]. We recruited participants from an academic primary care practice in San Francisco that serves a diverse group of > 24,000 patients. Participants were eligible if they were established patients in the practice, self-identified as Latino men, were $\ge$18 years old, preferred English or Spanish, had screened positive for depressive symptoms on the PHQ-2 (score $\ge$3) or had a self-reported or documented depression history, and had no prior diagnosis of bipolar disorder, schizophrenia, schizoaffective disorders, or dementia. Participants were recruited by N.S. and G.A.R. (see Acknowledgements) either (1) from a pool of prior study participants (inclusion criteria $\ge$40 years old, self-identified as Latino, preferred English or Spanish, and able to participate in a telephone interview) who had indicated interest in learning about future studies in the primary care clinic; [1, 68] or (2) were identified in the electronic health records (EHR) as eligible based on study inclusion criteria. We contacted the primary care physicians (PCPs) of any patient identified through the EHR to obtain permission to contact their patients. When recruiting participants, N.S. and G.A.R. described the study as one investigating men’s identities and depression. We initially used convenience sampling and subsequently switched to purposive sampling to identify and recruit monolingual Spanish-speaking participants (who were not initially well-represented among our participants). In this clinic, there are fewer monolingual Spanish-speaking Latino men compared to English-speaking Latino men, so we made a concerted effort to increase the language diversity of our sample. We recruited participants over the phone. Participants were recruited concurrently with data analysis, and recruitment was terminated once saturation was reached. Of 25 individuals who were eligible and reached, 13 participated (52%), 7 scheduled an interview but were lost to follow up or declined (28%), and 5 declined on initial outreach (20%). After the interview, participants completed a Patient Health Questionnaire-9 (PHQ-9) and a demographic questionnaire online (Table 1) [69]. Participants were encouraged to complete the PHQ-9 and demographic questionnaire immediately following the interview. Nine participants filled out the PHQ-9 the same day, one completed it the following day, and three participants took longer to complete the PHQ-9. We analyzed descriptive statistics in Excel.

### Semi-structured interviews

An interview guide was developed by N.S. and M.E.G. after reviewing literature on masculinity and depression from the fields of clinical psychology, psychiatry, critical masculinity studies, sociology of gender, gender studies, and history of medicine. Prior to initiating each interview, participants were provided with the opportunity to read through a summary of the study, the interviewer discussed the potential risks and benefits of engaging in the study, and each participant electronically signed a consent form. Participants were asked about their health and health care, experiences of depression, understanding and views of masculinity, and perspectives on barriers and facilitators to depression care. Immediately after the interview, each participant was mailed or emailed a $50 gift card. Interviewers (N.S. and A.C.F., full-time MD/MS students trained by M.E.G. MD, MPH, MAS) referred to the interview guide to ensure all subjects were discussed but asked questions in the order they arose organically in conversation. Interview guides were pilot tested on two Latino men who lived in the San Francisco Bay Area who did not meet study requirements (no documented history of depression) to maximize the number of eligible participants. Interview guides were not provided to participants. Questions were iteratively added over the course of the interviews to understand help-seeking processes, language barriers, the variety of treatments offered, and experiences with clinicians. Once written in English, A.C.F. translated the interview guide into Spanish, and M.E.G reviewed it (both native Spanish speakers). Interviewers conducted semi-structured interviews from December 2020 to August 2021 over Zoom or telephone and were audio-recorded. Field notes were made after interviews. N.S., a non-Latino white cisgender man, conducted all English interviews. A.C.F, a Cuban-American cisgender woman, conducted all Spanish interviews. Only the interviewer and participant were present during interviews. Interviewers had no established relationship with participants before study commencement. Participants were informed the study’s purpose was to explore barriers and facilitators related to masculinity that Latino men with depressive symptoms face in seeking depression care. This study was approved by the University of California, San Francisco Institutional Review Board.

### Qualitative analysis

Audio-recorded interviews were translated to English (when applicable), transcribed, and de-identified by a professional transcription service; all names are pseudonyms. We used an online qualitative analysis software, Dedoose (Los Angeles, CA), to code all transcripts. Coders (N.S., M.R., and M.L.) employed CQR to ensure coding was collaborative and egalitarian [65]. To create the initial draft of the codebook, all three coders individually coded the first manuscript, met to share their analysis, and collaborated on identifying, naming, and defining codes. This process was repeated for the subsequent three transcripts. After the fourth transcript was coded and the coders were in agreement about the codebook, coders would meet after coding 2–3 transcripts and would add to the codebook or further modify code names or definitions as needed. During these meetings, if any coders had questions or needed clarification about the codebook, the coders discussed and resolved these issues. M.E.G. served as the auditor, providing feedback on the codebook and helping to resolve discrepancies in definitions and application. Transcripts were coded concurrently with data collection to identify new themes and assess for saturation. Each transcript was independently coded by all three coders. The coding team discussed discrepancies and resolved them by consensus. We identified all barriers and facilitators, then categorized themes as related to masculinity if the participant (1) explicitly linked a barrier or facilitator to masculinity or (2) connected the barrier or facilitator to a component of masculine norms he or another participant discussed. Participants did not provide feedback on findings. As coders identified and defined themes, they began grouping them into domains, or broad categories that encompassed all barriers and facilitators and that allowed us to situate barriers and facilitators at different points on the spectrum of depression care. We created themes and domains iteratively: upon identifying new themes we would revisit the domains, and on revising our domains, we would sometimes modify our themes. We reached thematic saturation once three consecutive interviews yielded no new barriers or facilitators related to masculinity; data collection was terminated after the 13th interview met this criterion [70]. Select quotations in this paper are consistent with the remaining data.

## Results

We interviewed 13 participants, 10 in English and 3 in Spanish. The average interview length was 78 min (range: 37–141). The median PHQ-9 score was 10 (range: 0–24). While two participants did not endorse a depression diagnosis or have a PHQ-9 score $$\ge$$10 at the time of the interview, every participant had received recommendations by clinicians to engage in depression care due to a previous presentation with depressive symptoms. Additionally, all participants except for one (92.3%) received some form of depression treatment: nine participants (69.2%) utilized both psychotherapy and pharmacotherapy, and three participants (23.1%) engaged only in psychotherapy. Eight participants (61.5%) were first diagnosed with depression by their PCP. Eleven participants (84.6%) received referrals from their PCPs for specialized depression care. PCPs managed pharmacotherapy for three participants (23.1%). While all participants were recruited from a single primary care clinic, they discussed depression care they received throughout their lives in various clinical settings. The median age was 61 years (range 26–77). Six participants immigrated to the United States (four from Mexico, one from El Salvador, and one from Nicaragua) and one came from Puerto Rico. Three participants received disability benefits, eight earned less than $60,000 per year, and two earned more than $101,000 per year. All participants had insurance; 85% had Medi-Cal or Medicare and 15% had private insurance (Table 1).

**Table 1:** Participant Sociodemographic and Clinical Information (*N* = 13)

Characteristic	Median	Range
Age	61	26–77
PHQ-9*	10	0–24
	***N***	***%***
Cisgender man	13	100
Income, $		
Disability	3	23.1
10,000–20,000	4	30.8
20,000–30,000	2	15.4
30,000–60,000	2	15.4
> 101,000	2	15.4
Education		
Less than high school	5	38.5
Some college	4	30.8
Associate’s degree	2	15.4
Master’s degree	2	15.4
Country of origin		
Mexico	4	30.8
US	2	15.4
El Salvador	1	7.7
Puerto Rico	1	7.7
Nicaragua	1	7.7
Unspecified	4	30.8
Language of interview		
English	10	76.9
Spanish	3	23.1
Significant medical comorbidities	10	76.9
Has a diagnosis of depression	11	84.6
Depression self-recognition	11	84.6
Depression care		
PCP first to diagnose depression	8	61.5
PCP referred to specialized care	11	84.6
PCP managed antidepressants	3	23.1
History of psychotherapy and antidepressant use	9	69.2
History of only psychotherapy	3	23.1
No history of depression treatment	1	7.7
Current depression treatment	6	46.2

*PHQ-9: Public Health Questionnaire-9, self-administered assessment for the presence and severity of depressive symptoms. Ten participants filled out the PHQ-9 within one day of the interview. The three participants who responded later filled it out 8, 59, and 120 days after the interview.

**Fig. 1:**
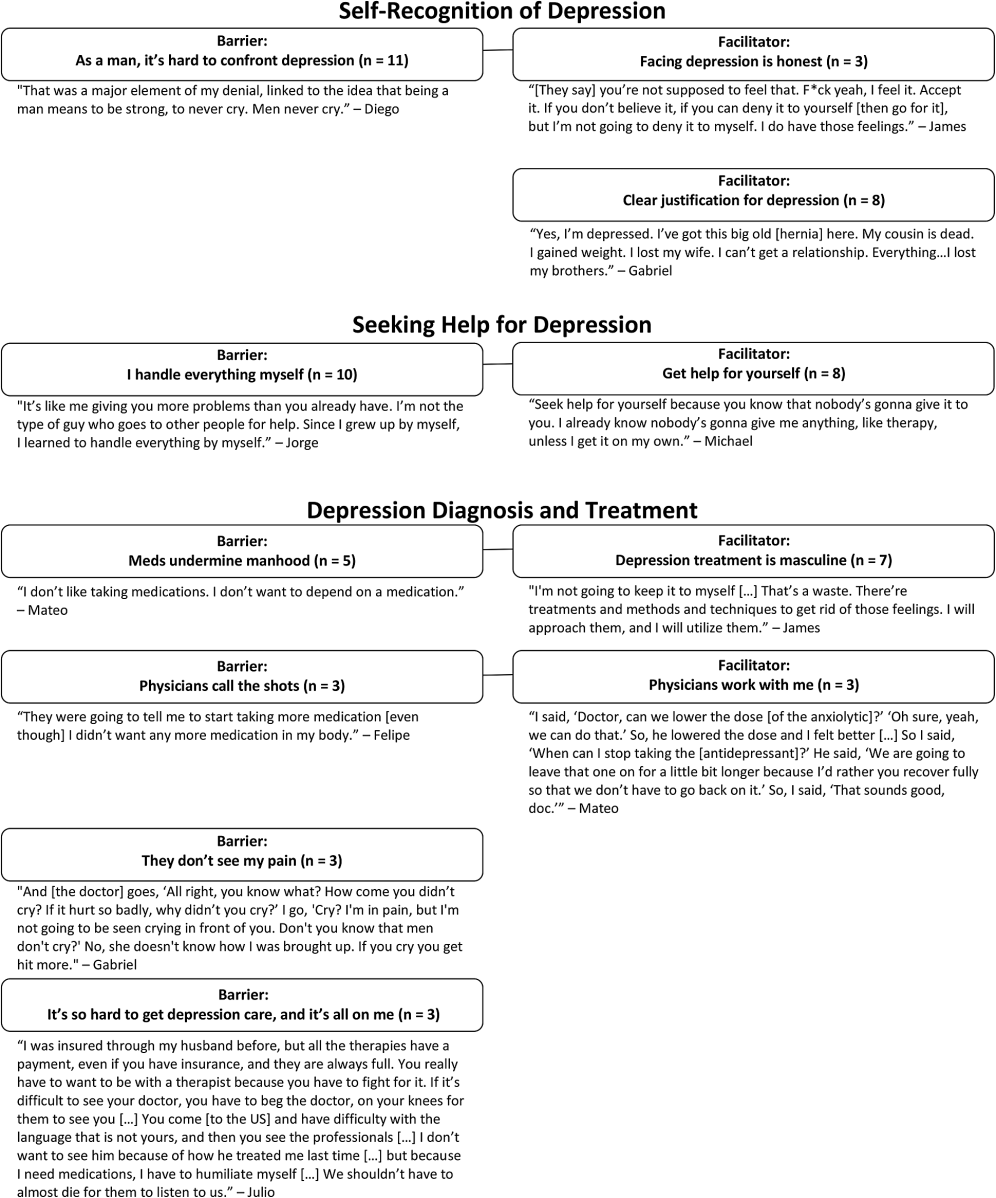
Barriers and Facilitators to Depression Care: Barriers and facilitators connected by a line are corollaries. The same values underly each, but they are expressed in opposite ways that either impede or promote depression care

Barriers and facilitators were organized into three domains: “Self-Recognition of Depression,” “Seeking Help for Depression,” and “Depression Diagnosis and Treatment” (Fig. 1).

### Self-recognition of depression

#### Barrier: as a man, it’s hard to confront depression

When asked, all but two participants elucidated ways depression created obstacles to upholding traditional masculine ideals and challenged conventional views of masculinity. Participants described their experiences of other people expecting them to maintain emotional control because they are men. James specifically identified the expectations he encountered as “*machismo”*:

“*Man up. A man is not supposed to do that or cry or feel those feelings. [The] stiff upper lip point of view: deal with it. You’re not sick. That’s all in your head.*” (James).

To the extent that participants were under external pressure to be stoic men, they said it was challenging to allow themselves to experience sadness, hopelessness, loneliness, and other vulnerable emotions associated with depression.

While James said he encountered this definition of masculinity from others but did not embrace it himself, many participants endorsed the importance of emotional control and paired it tightly with the central role of the man as the provider of the household. For Gabriel, to be a man meant to *“take care of your family. Protect them. Don’t cry. You’ve got to support your family.”* Participants discussed how depression undermined their ability to provide, rendering individuals unable to support their families, or worse, making participants feel like their families needed to take care of them.

*“For me what it means to be a man is first of all to be responsible, to be responsible for your family […] I don’t want to be a burden for my family, for my daughters, for my wife, because I’m the person who was responsible for supporting my family.” (Adriano)*.

Jorge never allowed himself to be provided for. Informed by his upbringing, he maintained that, without exception, men are the providers, not those who are provided for.

Men in my culture are the ones who provide for the household, not the other way around. [If you don’t], our culture is like, what’s wrong with you? You’re not providing. [I needed to] watch over [my] wife and at that time my child. It wasn’t easy. So, I had to learn to handle it.

Jorge handled his emotional distress himself, never engaging in depression treatment despite being urged to do so by a number of clinicians.

When asked whether he felt his ideas of what it meant to be a man contributed to his initial denial of a depression diagnosis, Diego replied, *“that was a big element of my denial […] We learn through media, TV, and other things that a man should be hard like a rock.”* However, even as Diego was trying to suppress his feelings of depression, he recalled recognizing the impossibility of realizing his ideals of masculinity.

When someone doesn’t cry, you become a timebomb. Repressed emotions are so bad. That was a major element of my denial, linked to the idea that being a man means to be strong, to never cry. Men never cry. Of course men cry. A real man is gonna cry.

Now that Diego has come to terms with his depression, he has redefined what it means to be a man in contrast to how he was raised and to the media he was surrounded by.

I don’t get that sh*t. That’s not a man. That’s a superhero. But real heroes have hearts, and real heroes cry, and their tears become diamonds. So, you want to be a rich man, cry as much as you want. Collect your diamonds. Wash your wounds, clean your heart now and then.

Diego demonstrates how people’s conceptions of what it means to be a man shift over time, particularly in response to direct experiences that challenge someone’s ideals of masculinity. Diego seemed to redefine masculinity to help himself make sense of his own experiences with and acknowledgement of his depression. For Diego, the stoic man is no longer the ideal. Rather, he felt he—and other men—should strive to embrace emotions.

#### Facilitator: facing depression is honest

Some participants identified honesty as a core component of masculinity. Although Michael believed that opening up about his history of trauma and emotional distress challenged his masculine identity, he ultimately defined what it means to be a man as telling the truth:

*I was molested [as a child], and there have been many painful situations in my life. I don’t want anyone knowing about that because it’s a private thing. I mean it makes me feel like less of a man. But I think that a man should definitely tell the truth at all times.*

Despite, or perhaps because, confronting histories of abuse and depression could be seen as emasculating, participants expressed that it took particular courage to be honest about their mental health. Participants framed courageous honesty as a value that affirmed masculine identity, and a facilitator for recognizing and accepting their own experiences of depression. Contrasting himself with other Latino men who are into “this machismo sh*t,” James portrayed acknowledging his own depression as honest.

[They say] you’re not supposed to feel that. F*ck yeah, I feel it. Accept it. If you don’t believe it, if you can deny it to yourself [then go for it], but I’m not going to deny it to myself. I do have those feelings.

Diego also viewed courageous honesty as a cornerstone of masculinity. “*A real man*” is not someone who stoically ignores his feelings, but “*someone who never hides his emotions. On the contrary, you develop courage to face what is, to face what you need to face.”* These participants viewed courageously facing the truth as part of masculinity and thus framed recognizing depression as masculine.

#### Facilitator: clear justification for depression

Many participants diagnosed with depression had a history of trauma and loss or a recent life-altering medical condition which they saw as more than a man could reasonably endure. They described their life circumstances as justifying depression. James, who suffered from a debilitating neurological disease, readily recognized his depression.

In my case, I’m not trying to figure out why I have depression. I know why I have depression. My medical conditions made me feel that way. I’m not searching for the reasons.

Similarly, Julio’s chronic medical conditions prevented him from being physically active, a major component of his identity before his illnesses.

I forget that I’m sick, and I want to do things. I can’t because I don’t have the energy. I want to run. I want to swim. I want to…but with these illnesses I can’t because I get very tired very quickly, especially with asthma. That depresses me because I feel like it cuts into my life. It’s sad because it’s aging me physically.

When a clinician at a community mental health clinic diagnosed Gabriel with depression, it immediately made sense to him because he was grieving for loved ones, got divorced, and was experiencing continued surgical complications.

Yes, I’m depressed. I’ve got this big old [hernia] here. My cousin is dead. I gained weight. I lost my wife. I can’t get a relationship. Everything…I lost my brothers.

Julio attributes his depression to his experiences of child abuse, his lack of English fluency, and being gay in a culture that rejected him.

I asked my mom, ‘Where were you when I was raped? What were you doing? Why did you let them? Where were you and my dad when I was abused? What did you do?’ […] Since you were abused as a child, everyone wants to take advantage of you, and you have to learn to stand up for yourself because I didn’t have an education. I didn’t know how to speak Spanish [properly]. I didn’t know how to write when I arrived here [in the United States], and then having to face English here, another language. I mean, that marks you. There are a lot of things that can lead to depression in a man like me, a gay man from a chauvinistic culture.

Participants rationalized depression as a logical response to traumas and medical illness, facilitating acceptance of a diagnosis they may have otherwise rejected because it challenged traditional masculine ideals and roles.

### Seeking help for depression

#### Barrier: I handle everything myself

Participants discussed their aversion to seek psychological help, referencing ideals of self-reliance and autonomy as well as expressing difficulty sharing emotions. Jorge emphasized the importance of self-reliance.

It’s like me giving you more problems than you already have. I’m not the type of guy who goes to other people for help. Since I grew up by myself, I learned to handle everything by myself.

While Jorge was on dialysis for years, he frequently lost hope, but he never sought mental health care, even when he was advised to do so by his doctors. Although he believed that therapists and psychiatrists could be helpful for others, he decided never to engage.

It’s a choice that you pick […] I never needed it. When you have God on your side and you are the type of guy who looks forward instead of looking at the problems, all those psychotic things don’t come into your head.

Jorge viewed himself as someone who could *“conquer any problem,”* so he didn’t need anyone to help him. His belief that engaging in depression treatment, and to some extent experiencing depression itself, was a choice reflects his efforts across decades to push through on his own.

Even those who, unlike Jorge, engaged in depression treatment expressed a desire to embrace self-reliance more fully. *“Eventually I’d like to wean myself off doctors. But for the time being, I still need them”* (James). Gabriel viewed seeking help for depression as especially difficult: *“It’s like being weak. It’s kind of embarrassing, looking for mental health.”* While he eventually sought and received depression treatment, his shame around getting help for depression delayed his engagement with care. Adriano also eventually sought help for depression from his PCP, but he was initially hesitant to even rely on his family to help him out because he viewed it as a form of *“begging.”*

I was a man who got everything I had myself. I would work for it, fight for it. I never had the hope that someone would give me anything.

Additionally, Adriano discussed the difficulty he experienced in being emotionally vulnerable. *“I was the person who kept everything in. Sometimes I would cry alone, but I didn’t know how to get it out.”* Difficulty expressing and identifying raw feelings and emotions is sometimes termed alexithymia and is more common among men than women [71–74]. Nicholas shared an almost total inability to talk about his feelings with others: *“It’s always been like this. I have never told anyone what I’m feeling.”* In order to communicate his feelings, James had to break down a *“barrier of keeping it inside”* over the course of several years.

#### Facilitator: get help for yourself

Conversely, participants who framed help-seeking as masculine saw it as self-reliant and responsible. While self-reliance is often viewed as precluding assistance from others, Michael stressed the need to “*seek help for yourself because you know that nobody’s gonna give it to you. I already know nobody’s gonna give me anything, like therapy, unless I get it on my own.*” Self-reliance was seen as encompassing relying on oneself to get help from others, framing depression help-seeking as the masculine choice.

Mateo advised other men like him who struggled with depression to ask themselves:

How do you feel about yourself? If you’re not okay with it, wouldn’t you want to feel better and try to understand what [help] is offered? Because if you never ask, you never get the help.

Like Michael, Mateo framed help-seeking as the self-reliant choice, because if you didn’t get help for yourself, who would? At different times in the interview, Mateo and other participants talked about how their spouses, children, and PCPs encouraged them to seek help. But at many points, participants framed their help-seeking as independent, self-reliant decisions. This may be due, in part, to many participants’ desires to frame themselves as autonomous, and to cast their decision to seek help as another way to rely on themselves.

In addition to viewing help seeking as self-reliant, participants also framed it as the responsible, strong, and proactive choice: a way to reclaim control of one’s life. Julio was

trying to be responsible. I’m trying to save my life. I’m trying to use extra strength, extra effort because not everyone does that. The easiest thing would be to go drinking, get drugged and die […] I have had to get serious and be courageous. I’ve had to love myself, to not let [depression] kill me, to not let it exhaust me. I will continue fighting.

After going back and forth on whether he should see a therapist for depression, Diego

reached a point where I said, okay, I need to take care of this. It’s really driving me crazy, putting my life out of control. If I continue like this, I’m just lying to myself. I’m not going to be able to overcome this.

Seeking help was a way for Diego to put his life back on track, to take control of a life in which he felt powerless. By saying, “*If I continue like this, I’m just lying to myself*,” Diego also illustrated how seeking help for depression can be intertwined with a more complete recognition of the existence and severity of depression. For some participants, denying that they needed depression treatment also meant denying their depression, whereas seeking out depression care was a further instantiation of acknowledging their depression.

### Depression diagnosis and treatment

#### Barrier: meds undermine manhood

For some, taking medications challenged masculine ideals of toughness, autonomy, and self-reliance. While most participants took pharmaceuticals for chronic medical conditions or depression, many “*don’t want to depend on a medication”* (Mateo). Although some participants ultimately decided to take antidepressants, they expressed their distaste for medications. Participants’ desire for autonomy and proclivity to ‘tough it out’ was amplified when they believed anti-depressants to be addictive—“*you get hooked*”—or irreversibly damage their bodies—“*nothing is temporary*”—heightening their sense of losing control (Felipe).

*“Sometimes I would lie that I had taken [the antidepressant] and I wouldn’t take it. Because for 55 years I never had to take a pill. I was never sick. So now I have 12 pills, 12 medications in the morning and eight at night. So there was a time when I said, ‘No, it’s too much medication.’”* (Adriano).

Adriano took pride in the fact that he was able to rely on himself for his health for the first five decades of his life. Now, faced with the onset of chronic physiologic conditions and depression, Adriano often chose not to take antidepressants because he did not want to rely on so many medications.

#### Facilitator: depression treatment is masculine

Many participants described receiving depression treatment as pragmatic and useful, identifying therapy as particularly practical, *“a simple solution to resolve”* depression (James), and a *“technique”* (James) to *“get it off your chest”* (Gabriel) and *“let things go”* (Adriano).

*“I’m not going to keep [my experiences of depression] to myself […] That’s a waste. There’re treatments and methods and techniques to get rid of those feelings. I will approach them, and I will utilize them.”* (James).

Adriano remembered guidance his psychologist offered that he continued to employ long after he stopped going to therapy:

Look, everything that you lost is already gone. Right now what’s important is that you are alive and you have a family who wants to see you. And if you want to see your family, you have to do your part.

Adriano’s psychologist successfully framed depression treatment as an active task that he should take to move forward, not back, thereby improving his own life. Michael found therapy useful because it provided a space for him to let out his feelings so that he could maintain his outwardly masculine behavior in settings where he felt less safe showing emotional vulnerability.

It was safe for me to tell her everything that was going on and not those guys back there because I was surrounded by people that just came out of prison. What are they gonna think about me? You know they’re gonna assume that I’m a weakling.

Some participants also described depression treatment using seemingly masculine metaphors: when a car is *“all out of oil and the engine is on fire,”* you need to *“fix it!”* (Mateo). By framing therapy in this language, participants may have been justifying their choices to take part in a process of understanding their mental distress as aligned with masculine ideals.

#### Barrier: physicians call the shots

Some participants felt physicians made unilateral depression treatment decisions, challenging their masculine ideals of self-reliance and autonomy. Participants expressed frustration and resisted treatment when they believed doctors provided them with insufficient medication information. Raul recalled when he identified one of the medications his neurologist prescribed as an antidepressant.

The funny thing is he didn’t really go over this medication, why he wanted to give it to me. The bottle was full. I never took one. In fact, I Googled what it was. It has very serious warnings. So, I’m glad I found it, and I flushed the whole bottle.

Because Raul did not believe he received sufficient information about the medication from his physician, he did his own research and disposed of the medication. Felipe felt like his PCP, psychiatrist, and psychologist tried to force him to take antidepressants. *“They tried really hard. And I kept telling them [no].”* Eventually Felipe agreed to try an antidepressant,

but then I found out later that a side effect of this medication was [a decrease in] my sex drive. Instead of making things better, it made it worse. So, I stopped taking that medication, and I stopped seeing that doctor.

Believing his clinicians had worn him down by repeatedly recommending medication and that his psychiatrist provided insufficient information about side effects, Felipe’s trust in his psychiatrist was broken, he stopped the medication, and he never saw that psychiatrist again. *“They were going to tell me to start taking more medication [even though] I didn’t want any more medication in my body.”*

Felipe and Raul both felt their clinicians forced their hand instead of inviting them to actively participate in their own care management.

#### Facilitator: physicians work with me

Especially regarding medications, a collaborative approach where participants felt included in treatment decisions throughout care was important to adherence. Mateo described how his PCP listened when he asked him to decrease the dose of his anxiolytic. When Mateo asked,

‘When can I stop taking the [antidepressant]?’ He said, ‘We are going to leave that one on for a little bit longer because I’d rather you recover fully so that we don’t have to go back on it.’ So, I said, ‘That sounds good, doc.’

These types of relationships affirmed patient autonomy, even when participants did not make every decision. Similarly, James appreciated the *“good rapport”* he has with his PCP. When James goes to the doctor with a concern,

We discuss it. We have a good relationship, a good discussion and conversation, and that helps. The way I approach my treatment for my illnesses is I go back home, I do my own research, and then when I go to see a doctor, I’m ready.

In contrast to Raul, who threw away his antidepressant after he did research on his own, James felt encouraged by his physician to learn about his options. His PCP actively including him in the decision-making process helped James feel more in control of his care and facilitated agreement in a treatment plan. While participants acknowledged the essential role that clinicians play in depression care, they often framed themselves as playing an active role in their own care: “*I’m trying to get myself better”* (Gabriel); *“I need to get ideas [from my PCP] as to how to handle this”* (Mateo); *“I don’t want to take medicine on my own, so I need to hear the doctor’s opinions”* (Raul). Participants emphasized the importance of patient agency in therapeutic decision-making, congruent with the masculine ideal of agency many described.

#### Barrier: they don’t see my pain

Participants recounted feeling their physicians took their physical and psychological pain less seriously because they viewed the participants as tough Latino men. After months of hearing that he weighed too much to undergo surgery for a hernia that was causing daily discomfort, Gabriel finally found a surgeon willing to operate. However, this surgeon seemed to disregard his pain.

Being a Latino man, I hate to say this, but my white surgeon who did my first surgery, she really treated me like I was some stupid man. [I said], ‘I’m trying to be nice to you and you are really rude to me.’ And then she said, ‘There’s nothing wrong, look.’ And she pushes my stomach back and forth. And I end up in the emergency room the next day. I told her, ‘Don’t do that, because every time you shove my stomach around, it upsets my whole stomach and I get sick’ […] And then she had her and her colleague pushed my [stomach] around. And she goes, ‘All right, you know what? How come you didn’t cry? If it hurt so badly, why didn’t you cry?’ I go, ‘Cry? I’m in pain but I’m not going to be seen crying in front of you. You know that men don’t cry.’ No, she doesn’t know how I was brought up. If you cry you get hit more.

Gabriel thought that his surgeon perceived him as tough and less likely to experience pain because he was a Latino man. Additionally, he maintained his surgeon did not trust him when he said he felt pain because he did not express his pain in a way she recognized. Gabriel attributed his stoicism to physical pain to his upbringing, where he learned not to cry to uphold masculine ideals and avoid physical punishment. Gabriel believed that his surgeon mistreated him specifically due to her perception of Latino men.

Stefano recounted experiences where clinicians found it difficult to recognize not only his physical, but also his mental pain.

*When people look at me, they just see a big guy. And they don’t realize that I do have physical and mental and vestibular issues going on.*

#### Barrier: it’s hard to get depression care, and it’s all on me

Participants’ attitudes toward engaging in depression care were also impacted by structural barriers to treatment: short appointments, care team discontinuity, few mental health providers, no insurance coverage for preferred treatments (e.g., psychotherapy), language barriers, mistreatment, and racism. These barriers amplified difficulties posed by masculinity and placed our participants in vulnerable situations where they felt they needed to compromise their ideals of autonomy, self-reliance, and dignity. Even though Julio was insured through his husband,

all the therapies have a payment, even if you have insurance, and they are always full. You really have to want to be with a therapist because you have to fight for it. If it’s difficult to see your doctor, you have to beg the doctor, on your knees for them to see you […] [People like me,] we have psychological problems. Since we were small, we were physically abused, mentally abused, violated, and then we come [to the US] and have difficulty with the language that is not yours […] I don’t want to see [my PCP] because of how he treated me last time […] but because I need medications, I have to humiliate myself […] We shouldn’t have to almost die for them to listen to us.

All participants who wanted depression care discussed difficulties in receiving it, but Julio illustrates how a multiplicity of factors—his history of trauma, treatment unaffordability and limited availability, language barriers, and medical mistreatment—compounded to make him feel humiliated and vulnerable, rendering his continued engagement with depression treatment almost unbearable.

## Discussion

Participants described ways masculinity influences barriers and facilitators to depression care at three stages: self-recognition, seeking help, and diagnosis and treatment. At the stage of self-recognition, while participants shared the belief that men should not feel depressed, they also described facing depression as masculine because it was honest. Some participants chose not to seek help, wanting to handle depression themselves, while others justified their decision to seek help as self-reliant. Participants described barriers to diagnosis and treatment when they experienced apparent discrimination from physicians or felt their autonomy challenged and emphasized how collaborative patient-clinician relationships encouraged treatment continuation. Our findings support recent research emphasizing that while masculinity likely contributes to underdiagnosis and undertreatment of men’s depression, [21, 22, 36, 40, 46, 75, 76] different components of traditional Western masculine norms may encourage depression care engagement [48, 51–53]. 

This paper adds to the literature by eliciting and examining the real-life choices that Latino men made about how they wanted to engage in depression care. Our results help to expand understanding of how Latino men negotiate their values, how their ideas of masculinity shift over time and context, and how they make decisions in various clinical settings with different health care professionals. These findings could therefore inform clinical approaches that aim to minimize the barriers and maximize the facilitators to depression care that our participants have faced.

While some participants did not currently endorse depressive symptoms as determined by their current PHQ-9 scores, all had a history of clinicians identifying a presentation of depressive symptoms. Additionally, some PHQ-9 scores—particularly those that were 0—may not accurately reflect our participants’ symptoms. Participants may have hesitated to recognize or explicitly endorse depressive symptoms [21, 22, 77]. Significantly, our sample included participants who did not believe they suffered from depression and who declined services, despite clinicians encouraging them to accept depression care. This study contains perspectives from people who are rarely represented in the literature on masculinity and depression: those who rejected their diagnoses of depression and declined depression treatment [21, 22, 51, 54]. Our participants shared a uniquely wide array of experiences, ranging from no engagement with depression treatment to extensive and continued engagement. Understanding the reasons and motivations underlying why some Latino men engage in depression care and others do not is essential to addressing mental health care disparities among Latino men.

Some participants discussed machismo and gendered racism as barriers to depression care. Many described how the ideal they were raised with—of a man who handles everything, provides for his family, and doesn’t let anything get to him—created unique obstacles for them as Latino men. Some of our participants embraced these macho values, but many distanced themselves from them, defining their masculine identities in opposition to machismo. Since all our participants were Latino, we are unable to make claims about the degree to which Latino conceptions of masculinity differ from white conceptions, only that some participants viewed the masculine ideals they grappled with as culturally unique. Participants also recounted experiences of gendered racism wherein they believed physicians disregarded their pain, viewing them as tough Latino men. Gendered racism occurs when stigma related to race or ethnicity and gender “intertwine and combine under certain conditions into one, hybrid phenomenon.” [78] Participant reports of gendered racism demonstrate important ways that ideas about race and ethnicity may shape clinicians’ perceptions of men, even in clinical settings [59, 61–64, 79, 80]. While participants discussed machismo and gendered racism that were unique to their experiences as Latino men, they also described many aspects of masculinity that were identified in studies among white men. This is consistent with previous studies which reveal certain differences in how Latino and white men discuss gender and depression such as Latino men stressing the importance of providing for their families and white men emphasizing individual economic self-sufficiency. However, both Latino and white men expressed similar masculine norms such as pressure to provide and be productive, autonomy, self-reliance, strength, responsibility, and social status [81, 82]. The differences thus appear to be not in the content of the masculine norms they discuss—which seem to be shared—but rather the relative importance of the masculine values and ideals and how frequently these are highlighted.

We identified components of masculinity that were associated with barriers—emotional control and the pressure to provide—and facilitators—honesty, courage, practicality, responsibility—to engaging in depression care. Self-reliance and autonomy sometimes promoted and other times discouraged help-seeking. While quantitative studies consistently associate increased self-reliance with decreased depression help-seeking, our study elucidates a definition of self-reliance that facilitates seeking help for depression care [10, 36, 50]. This contrast between *I handle everything myself* and *Get help for yourself* illustrates how self-reliance can be framed as a motivation for refusing or engaging in depression care. Clinicians could thus view self-reliance not as a pathologic proclivity to refuse all aid, but rather as an important component of a person’s identity that can help patients to motivate themselves to take control of their life by relying on themselves to get care. Though this process can take years or decades, many participants found a way to portray their decisions as affirming masculine ideals, or to redefine masculinity. While some of the themes (e.g., *As a man, it’s hard to confront depression* and *Depression treatment is masculine*) initially appear contradictory, this may reflect the nuanced and sometimes conflicting ways that masculinity can affect depression and help-seeking. These tensions and shifts in the application of masculinity point toward masculinity as a multitudinous, flexible set of ideals that can be applied in different ways in different times and contexts [19, 22, 48, 51, 54]. Instead of viewing masculinity as a homogenous, rigid set of expectations that only create barriers to depression care, masculinity is more accurately understood as a wide array of values that can be shaped by circumstance and situation [19, 22, 51]. 

Our results suggest some steps clinicians could take to frame depression care as affirming masculine identity to facilitate engaging in depression care. We found four barriers and facilitators that were complementary, deriving from the same underlying value (Fig. 1). Clinicians could identify the barriers and facilitators their patients experience at the stages of self-recognition, seeking help, and diagnosis and treatment, and encourage patients to frame their values in a way that promotes depression care instead of inhibiting it. For example, for men who are struggling to recognize their depression because it contradicts their ideals of masculinity, clinicians could frame confronting depression as honest and courageous. For those having trouble seeking help, physicians could portray help-seeking as self-reliant, and for men who believe antidepressants challenge autonomy, clinicians could emphasize how medications can help patients regain control.

PCPs could further enhance men’s engagement in depression care by employing shared decision making to encourage collaborative patient interactions. Shared decision making—an approach wherein the clinician and patient both share their knowledge, goals, and preferences and work together to arrive at a medical decision—has been shown to improve patient health outcomes, treatment adherence, patient satisfaction, healthcare empowerment, and trust in clinicians [83–85]. Our results suggest that shared decision making and clear clinician communication may be particularly important to maximizing the chance that Latino men engage in depression care. By engaging patients in a collaborative, bilateral conversation, PCPs can take advantage of the facilitator *Physicians work with me*, affirming their patients’ values of autonomy and control, and minimize the chance of creating the barrier *Physicians call the shots*, where patients feel out of control and uninformed. Some of the participants who decided to continue taking antidepressants appreciated how their clinicians treated them as active collaborators in their own care. In these instances, patients felt clinicians explicitly valued their opinions, and shared their own recommendations informed by their clinical knowledge. Conversely, participants who decided to discontinue depression treatment felt their clinicians made unilateral decisions without sufficiently eliciting or understanding their preferences or values. Shared decision making could be an essential approach that would both help Latino men feel like a partner in creating a care plan and that would help PCPs understand the specific motivations of each of their patients.

When men are hospitalized or receive a life-altering diagnosis, physicians should assess mental health and screen for depression. The diagnosis of grievous medical conditions may create unique opportunities since some men—like our participants—may be more amenable to recognizing depression when they believe it is justified by external factors. PCPs could capitalize on this opportunity by specifically integrating assessment for depressive symptoms in their workflow when their patients are discharged from the hospital or if they receive life-altering diagnoses. PCPs are uniquely positioned to screen for depressive symptoms both because they are more likely to have a longitudinal relationship with patients and because timely outpatient follow-up is routinely scheduled after extended hospital stays. Routine mental health screenings may also alert clinicians to depressive symptoms they may have otherwise overlooked, as studies indicate clinicians recognize depression less often in men [46, 75]. 

Our findings could also inform education and training for clinicians on how gender can influence patient presentations and experiences with depression and treatment. Gender should be understood as influencing *how* different people relate to and experience depression, not as a determinant of *who* experiences depression. Approaches that recognize the barriers and facilitators shaped by gender should emphasize understanding each individual’s values and experiences, how they could be affected by gender, and how to affirm those values through depression treatment. Training and education that specifically focuses on the intersection of gender, race and racism, and depression care could serve to reduce clinician bias, foster an inclusive clinical environment for all patients, and optimize depression care engagement.

Finally, Latino men who face barriers related to masculinity also contend with enormous structural barriers to depression care that must be addressed: few mental health care providers and appointments, care team discontinuity, prohibitive costs, healthcare bureaucracy, and language barriers [1, 7, 49]. As the barrier *It’s hard to get depression care, and it’s all on me* demonstrates, barriers to depression care related to masculinity do not exist in isolation. Our participants described ways that these structural barriers made them feel powerless and vulnerable, challenging their identity as men. Thus, barriers to depression care shaped by masculinity could exacerbate various structural barriers.

### Limitations

During recruitment, participants were told this study explored depression and masculinity; our participants may have been more comfortable discussing these topics than individuals who declined to participate. All participants lived in the San Francisco Bay Area, a region of the United States that is more politically liberal than average, so the way our participants discussed masculinity, depression, and depression care may also have been skewed toward more open and accepting views than exist more broadly throughout the country. Recruitment of Spanish-speaking patients began later, and fewer Spanish speakers participated prior to study saturation, limiting claims we can make about monolingual Spanish-speaking Latino men. While the varied interviewer sociodemographics and positionality could have affected interview responses, we believe this impact was minimal given the overlap in themes identified between interviewers and the level of detail that participants shared in all interviews. Participants were recruited from one primary care practice in a large, academic health system, and they were older, which may have increased the proportion of participants with serious comorbidities. However, this also provided a unique window into the barriers and facilitators medical conditions posed to depression care and the variety of interactions between patients and clinicians, making these results more generalizable to primary care settings. In order to focus specifically on depression care and to minimize chances that impaired memory or perception interfered with interview responses, people with bipolar disorder, schizophrenia, schizoaffective disorders, and dementia were excluded. This limits what we can generalize about barriers and facilitators to depression care among Latino men with these comorbidities. All participants were currently insured, limiting this study’s ability to identify barriers related to lack of insurance, although some participants did recount instances when they were previously uninsured.

## Conclusion

Gender norms shape important barriers and facilitators for Latino men with depressive symptoms at the levels of self-recognition, seeking help, and diagnosis and treatment. We identified specific components of masculinity that were associated with barriers, facilitators, and both. To counteract underdiagnosis and undertreatment of depression among Latino men, PCPs and other clinicians could understand patients’ values and experiences, framing depression care as masculine. Providing education to PCPs and other healthcare professionals on gender and depression—to reduce clinician bias, foster an inclusive environment for all patients, and optimize depression care engagement—and addressing structural barriers are essential to providing access to all who need depression care.

### Electronic supplementary material

Below is the link to the electronic supplementary material.

Supplementary Material 1

## Data Availability

The de-identified qualitative datasets used and analyzed during the current study are available from the corresponding author on reasonable request.

## Abbreviations

CQR: Consensual Qualitative Research
EHR: Electronic Health Records
MDD: Major Depressive Disorder. PCP:Primary Care Physician
PHQ-2: Patient Health Questionnaire-2
PHQ-9: Patient Health Questionnaire-9
