# Supplementary material for: Barriers and facilitators to depression care among Latino men in a primary care setting: a qualitative study
Source: BMC Prim Care. 2024 Jan 20;25:30. doi: 10.1186/s12875-024-02275-x (PMC10799470; doi:10.1186/s12875-024-02275-x)
Supplement: Supplementary file 1 — Supplementary Material 1 [file 12875_2024_2275_MOESM1_ESM.docx]

Supplementary Material

**Interview Guide – English***

Domains

1. General health and healthcare practices (5 minutes)
   1. Before COVID, how often would you say you come in for a doctor’s appointment each year?
      1. After COVID, how has this changed?
   2. Could you tell me about the sorts of things that generally bring you in for an appointment?
   3. How has COVID-19 impacted your recent decisions regarding your health and seeing the doctor, either using telehealth or going in person?
   4. Could you talk about what’s been bringing you in for appointments recently?
   5. Could you describe a bit more about your past medical history?
   6. Continuity of care?
2. Experiences with depression (10 minutes)
   1. Could you talk about how your mood has been recently?
      1. What has been contributing to that?
         1. Current changes due to COVID-19?
            1. Job instability
            2. Sheltering in place
            3. Death of family/friends
            4. Worries about the future
      2. How would you say that makes you feel?
      3. How would you define how you are feeling?
         1. Would you classify your feelings as depression?
            1. Could you tell me a bit more about why not?
            2. Could you tell me a bit more about why you would?
   2. Alt: One of the reasons you were contacted to participate in this study is because you screened positive for depressive symptoms at one of your primary care appointments. Are you still experiencing any of those symptoms?
      1. Depressed mood
      2. Less interest
      3. Weight loss
      4. Low energy
      5. Trouble sleeping or sleeping too much
      6. Worthlessness/guilt
      7. Loss of appetite
      8. Low concentration
      9. Thoughts of death and dying
      10. Anger
      11. Stress
      12. Guilt – as distinguish from medical conditions
   3. Have you had discussions about your symptoms with your doctor?
   4. How did you go from experiencing depression to receiving treatment for it? What did that process look like?
   5. What feelings came up when you were diagnosed with depression/discussed depression?
      1. Relief/rejection?
      2. What was it like for you to fill out the depression scale the doctor gave you in primary care?
      3. Were you diagnosed because you brought it up or were you screened for depression?
         1. If you were screened, how did that feel for you?
   6. How did you feel about the treatment that was offered to you? What feelings came up around your treatment decision?
      1. What sort of treatment have you been offered?
      2. What does self-treatment look like for you?
      3. What treatment have you used that the doctor didn’t prescribe? Traditional healing? Curanderos?
      4. What do you think was missing in the treatment that was offered to you?
      5. What role do sleep, exercise, diet, and socializing play in managing your depression?
   7. You mentioned you’ve been generally feeling ____. When you feel like that, what does your day generally look like?
      1. Could you tell me a bit more about how that feels?
   8. How do these feelings impact how you interact with other people in your life?
      1. Family
         1. Spouse
         2. Kids
         3. Parents
      2. Work
         1. Coworkers
         2. Bosses
         3. Employees
      3. Social
         1. Friends
         2. Friend groups
         3. Social/religious institutions
      4. Strangers
   9. When you’re depressed who are you interacting with? Are these people helpful when it comes to depression, or do they make things worse?
3. Self-definition of masculinities (10 minutes)
   1. Could you talk about how your recent experiences with mental health have impacted how you see yourself?
      1. Impacted how you see yourself in relation to others around you?
      2. Changed the way you see yourself?
   2. You mentioned your depression/sadness/loneliness/anger has impacted ____. How would you say it’s impacted how you see yourself as a man?
   3. How would you define what it means to be a man?
      1. When you think of what it means to be a man, what comes to mind?
      2. Could you talk about a couple of ways you try to be a good man? (Alt: when you think of what makes somebody a good man, what comes to mind?) (Alt: when you think of men who were role models for you, what comes to mind?)
         1. Who have been figures in your life who modeled ways of being a man that you hope to emulate?
         2. What are some components of masculinity that you find to be positive and affirming?
      3. What are some things that come to mind when you think of bad ways to be a man? (Alt: when you think of what makes somebody a bad man, what comes to mind?)
         1. What about ways that you would think of yourself or others as less of a man?
            1. Are those the same things?
   4. What does it mean to you to be a Latino man?
4. Response to traditional categories of masculinities
   1. You’ve talked about how your idea of what a man is involves ____. To what extent does your idea of manhood relate to having a family?
      1. Dating?
      2. Sex?
      3. Having a job/supporting a family?
         1. breadwinner
   2. How does it relate to emotions you’re feeling?
      1. Sharing those emotions?
      2. How important is it to you to keep your emotions to yourself?
         1. To deal with your feelings on your own?
      3. Individualism, stoicism, self-reliance, toughness
   3. Competition/status
   4. Risk taking
   5. Violence/aggression
   6. Relationship to women (power)
   7. (hetero)sexuality
5. View of structural/attitudinal barriers/facilitators
   1. Do you have any friends who are dealing with depression?
      1. Do you know the sort of treatment they’re receiving?
      2. Could you tell me a bit about what you think about that treatment?
   2. You talked about how you screened positive for depression at your primary care clinic. Have you thought about care?
      1. If so, what type of care?
      2. To what extent do you feel like you would want care? Need care?

There are a number of things that make it harder or easier for people to seek out care for depression. I’d like to talk a little bit about both what has made it difficult and what has made it easy in your experience.

- 1. Could you tell me about some of the things that have stood in your way to seeking out care specifically for depression?
     1. Clearly you speak English is great. Was that always the case? Has that happened with you in depression care?
     2. Sometimes language barriers impact the way people can talk about their experience. Has that happened
     3. To what extent do you feel like you have the tools to seek out mental healthcare?
        1. What are some issues that are still unclear that stand in the way?
        2. In the COVID-19 era of telemedicine, how equipped do you feel to seek out mental health services?
     4. Could you talk about the extent to which affordability is a factor?
     5. When you see your primary care physician, how comfortable do you feel talking about your mental health?
        1. Do you feel like talking about mental health is prioritized by your provider?
        2. Do you feel like you have enough time to fully answer questions that your primary care physician asks you about your mental health?
        3. How much would you say you trust your healthcare providers?
           1. Some trust more than others?
           2. Poor medical experiences?

Many people experience racism in the medical system. In what ways has racism or racist actions impacted your care or the way you’ve been treated?

- - - 1. How much does it seem like your providers know about mental health? Want to talk about it?
    1. Continuity of care?
    2. Are there any hesitancies you have about seeking out care for depression or mental health more broadly?
       1. Could you tell me a bit more about the personal obstacles that stand in the way?
          1. Self-image/identity/masculinity
          2. Time constraints
          3. Not on your mind
       2. How do you think seeking out care would impact how you see yourself?
          1. How does your current situation impact how you see yourself? (pull in what they’ve said about masculinities; opp to draw on previous responses)
       3. What do you think would happen if you sought out therapy/treatment for depression?
  1. What have been some things that have made it easier for you to take steps to seek out care for depression? (*I won’t be asking each of these, but again, a good list of a variety of options to have on hand*)
     1. Social support?
        1. Family
        2. Friends
     2. Masculinity/personal motivation
        1. Self-reliance/independence
        2. Bread winning
        3. Attitude toward help seeking
     3. Providers?
        1. Warm hand offs?
     4. Community organizations?
     5. Financial/accessibility?
     6. Gaining knowledge?
     7. Telemedicine/fewer COVID barriers?
     8. Media/ads promoting mental health awareness and treatment?
     9. Level of severity?
     10. Positive experiences in the past with mental healthcare?

1. How have you overcome obstacles and utilized facilitators to seek out care, or how would you do so?
   1. Since you screened positive for depression at your appointment on ____, have you followed up with specific appointments addressing depression?
      1. If yes
         1. Looking back, what do you think were the most important things that motivated you to seek out further mental healthcare?
            1. Could you tell me a bit more about what that process was like for you?
            2. Were there any people in your life who particularly encouraged you to seek out care, or helped you in some other way?
         2. You’ve already done ____. Do you see yourself continuing to seek out this sort of care?
            1. What sorts of things motivate you to continue seeking out this sort of treatment?
            2. Do you have any hesitancies?
            3. Even though you’ve sought out this care already, are there still things that make it difficult?
      2. If no
         1. Could you talk about how you decided not to seek out care?
            1. Is seeking out care something you would be interested in doing?
         2. What are some things that would motivate you to seek out care?
         3. In an ideal situation, how would you want your depression treated?
            1. How do you think you could get some of that treatment given the constraints we have?

Is there anything we haven’t touched on that you wanted to bring up?

**Spanish version available upon request. Please email nate.swetlitz@ucsf.edu.*
